# Comparative Efficacy of Oral Apixaban and Subcutaneous Low Molecular Weight Heparins in the Treatment of Cancer-Associated Thromboembolism: A Meta-Analysis

**DOI:** 10.7759/cureus.43447

**Published:** 2023-08-14

**Authors:** Maryam F Baloch, Adedimeji V Adepoju, Vaibhavkumar Falki, Mohsin Hajjaj, Tatiana Habet, Karina Habet, Amtul Mahrosh, Sumana Kundu, Janvi Kataria, Midhun Mathew, Tugba Saka, Mohammed Al-Tawil

**Affiliations:** 1 Department of Community Medicine, Allama Iqbal Medical College, Lahore, PAK; 2 Department of Medicine, Oak Hill Hospital, Brooksville, USA; 3 School of Medicine, Corewell Health University Hospital, Grand Rapids, USA; 4 Department of Internal Medicine, Jinnah Hospital Lahore, Lahore, PAK; 5 School of Medicine, Universidad de Ciencias Médicas, San Jose, CRI; 6 School of Medicine, American University of Antigua, Antigua, ATG; 7 School of Medicine, Dr. VRK Womens Medical College, Hyderabad, IND; 8 Department of Research, R.G. Kar Medical College and Hospital, Kolkata, IND; 9 School of Medicine, DY Patil University, Mumbai, IND; 10 Department of Internal Medicine, Pennsylvania Hospital, Philadelphia, USA; 11 School of Medicine, Istinye University, Istanbul, TUR; 12 Department of Surgery, Al-Quds University, Jerusalem, PSE

**Keywords:** randomized, thromboembolism, cancer, lmwh, apixaban

## Abstract

Cancer patients' risk of developing venous thromboembolism (VTE) is four to seven times higher than the general population. Cancer-associated VTE (CA-VTE), is a leading cause of morbidity and mortality in cancer patients. Low Molecular Weight Heparin (LMWH) has historically been the mainstay treatment of CA-VTE; however, complications such as bleeding and recurrent VTE make it challenging to manage these patients. Recent randomized controlled trials (RCTs) have proven that direct oral anticoagulants (DOACs) are as efficacious as LMWHs in treating CA-VTE. We conducted a systematic review and meta-analysis to ascertain the efficacy and safety of LMWH and Apixaban for the treatment of CA-VTE. A systematic review was conducted using Medline, Embase, and Scopus, databases for all cohort studies, case-control studies, and RCTs in English comparing cancer patients undergoing treatment with Apixaban or LMWH to treat CA-VTE from inception-May 2023. The Review Manager program, version 5.4.1, was used for statistical analysis and the Mantel-Haenszel fixed-effects models to calculate the risk ratio (RR) and 95% confidence intervals (CIs) and the inverse variance approach to get the weighted mean difference for the continuous outcomes. Q-test for heterogeneity was used to examine statistical heterogeneity and an I^2 ^statistics value >50% was defined as significant heterogeneity. A total of four studies were included, and the total number of patients was 1,632 across all studies. The Apixaban group was associated with a statistically significant increase in minor bleeding (RR 1.57; 95% CI (1.12, 2.21); p=0.009; I^2^=0%), but not for major and total bleeding. The Apixaban group showed a statistically significant lower risk of recurrent VTE when compared to the LMWH group (RR: 0.61; 95% CI (0.41, 0.92); p=0.02; I^2^ = 7%), and there was no statistically significant difference in terms of mortality between the two groups (RR: 0.89; 95% CI (0.73, 1.09); I^2^=0). Our findings suggest that Apixaban may be a favorable anticoagulant option for managing cancer-associated thromboembolism, as it demonstrated a lower risk of recurrent VTE. The risk of bleeding with DOAC in gastrointestinal cancers warrants further investigation.

## Introduction and background

Cancer is a significant risk factor for the development of venous thromboembolism (VTE), including pulmonary embolism (PE) and deep venous thrombosis (DVT) [1]. Patients with active cancer face a four to sevenfold higher risk of VTE than those without cancer [1-3]. This accounts for about 30% of VTE cases. Cancer-associated VTE (CA-VTE), which includes DVT and PE, is the leading cause of morbidity and death in cancer patients [4]. Several factors contribute to the development of CA-VTE. These include tumor-associated factors, treatment-related factors such as surgery, chemotherapy, hormonal therapy, stage of cancer, type of cancer, and patient characteristics like obesity and advanced age [1,5,6]. This risk is further increased by patient comorbidities especially gastrointestinal, pulmonary, and cardiovascular diseases [3]. 

Current guidelines recommend anticoagulant therapy for a duration of six months in most cancer patients [1,2]. Historically, LMWH is preferred to Warfarin for the treatment of CA-VTE [3]. However, treatment with LMWH for more than six months may cause several adverse effects, such as bruising or pain [1]. DOACS (Rivaroxaban, Edoxaban, Apixaban) are oral direct Xa inhibitors. Apixaban is typically administered in fixed doses to treat CA-VTE for a duration of six months [6]. This helps to mitigate the hypercoagulability state caused by the procoagulants released by tumor cells.

In recent years, randomized controlled trials (RCTs) have been conducted, demonstrating that direct oral anticoagulants (DOACs) are as effective as LMWH for the treatment of CA-VTE and are more convenient for patients [1]. In an RCT conducted in 2020 that compared the efficacy and safety of apixaban and dalteparin (an LMWH), apixaban demonstrated superior results [1]. This finding and similar evidence for other DOACs, such as rivaroxaban and edoxaban, support using DOACs as alternatives to LMWH based on their efficacy and safety for VTE treatment in individuals with cancer [7-9]. These studies have led major international organizations to consider DOACs as alternatives to LMWH to treat CA-VTE.

In light of these developments, the aim of this review is to examine the evidence regarding the use of apixaban (a DOAC) and LMWH in the treatment of cancer-associated thrombosis (CAT). The research primarily seeks to compare the efficacy and safety of Apixaban, a DOAC, and low molecular weight heparin (LMWH) in managing CA-VTE. It concluded that Apixaban might be a favorable option for managing CA-VTE as it demonstrated a lower risk of recurrent VTE. However, there was a statistically significant increase in minor bleeding associated with Apixaban. The research fits into the ongoing scientific debate about the best anticoagulant option for CA-VTE patients. The study presents a systematic review and meta-analysis of previous RCTs comparing Apixaban and LMWH, thus contributing valuable synthesized data to the existing body of literature.

## Review

Methods

Literature Search

Medline, Scopus, and Embase were used to identify the relevant studies in this systematic review and meta-analysis. We included studies published from each database inception date up to May 26, 2023. We applied the following search strategy to retrieve articles mentioning the following search terms in their title/abstract (Apixaban OR Eliquis) AND (Low Molecular Weight Heparins OR LMWH OR Dalteparin OR Enoxaparin OR Tinzaparin) AND (Cancer OR Malignancy OR Neoplasm OR Metastatic growth OR Tumor) AND (Thromboembolism OR Embolus OR Thrombus OR Blood Clot) AND (Randomised OR Randomized). In addition to searching the databases, we also utilized a backward-forward citation check to identify additional RCTs from the references of relevant articles.

Inclusion and Exclusion Criteria

The initial database search for relevant studies was performed by one investigator. Four investigators independently performed title-abstract and full-text screening using the Rayyan platform to identify eligible studies; any conflicts were resolved by another reviewer. The inclusion criteria used for the comprehensive searches were cohort studies, case-control studies, and RCTs (in English) with at least 50 cancer patients who underwent therapy with Apixaban or LMWH to treat cancer-associated thromboembolism as the primary/ initial treatment and reported either one or all of the following treatment outcomes - major bleeding, clinically relevant non-major bleeding (CRNMB), total bleeding, any recurrent VTE, and all-cause mortality. We excluded studies that are non-English, systematic reviews, meta-analyses, narrative reviews, case reports/ series, editorials, study protocols, abstracts, commentaries, letters, and the studies which document the outcomes for only one treatment for example: outlining the framework for Apixaban therapy and discussing its outcomes only without drawing a comparison with LMWHs.

**Figure 1:**
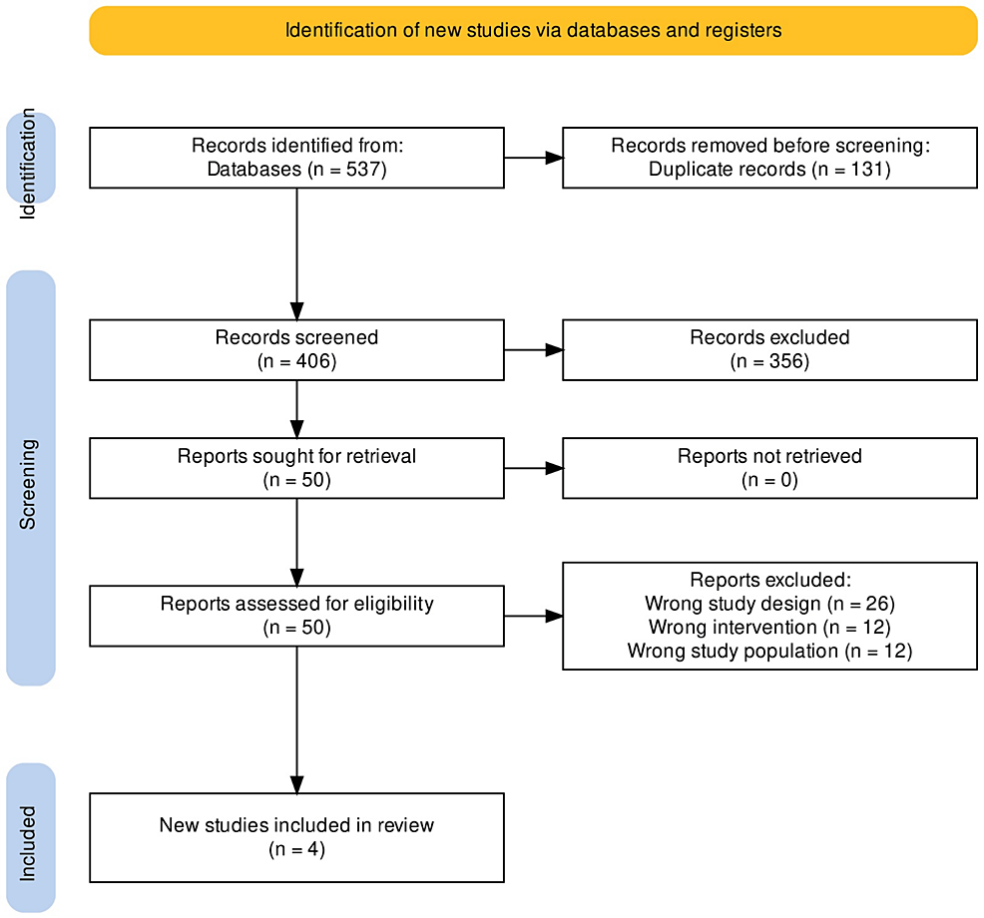
Flow chart describing systematic search and study selection process

A total of 537 articles and abstracts met the initial search criteria. Of these, 131 duplicates were removed, leaving 406 records to be screened. The studies for this meta-analysis were excluded based on specific criteria to ensure the inclusion of high-quality and relevant evidence. Firstly, studies were excluded if they failed to provide an effective comparison between apixaban and LMWH, as the direct comparison of these treatments was crucial for the analysis. Additionally, any studies not meeting the predefined inclusion criteria, such as those not being RCTs, were excluded to maintain the integrity and validity of the findings. Moreover, studies focusing exclusively on women were excluded to ensure a more diverse and representative study population. Lastly, studies with insufficiently discussed or reported outcomes related to apixaban and LMWH were also excluded, as the availability of comprehensive outcome data is vital for drawing accurate and reliable conclusions. Four studies were included in the meta-analysis after four researchers' full-text assessment of 50 articles and conflict resolution by two others. All other studies were excluded due to either the study design (n=26), intervention (n=12), or study population (n=12), not meeting the inclusion criteria as depicted in Figure 1*.* All four included studies were randomized controlled studies [10].

Data Extraction and Quality Assessment 

The construction of the data-extraction Excel sheet was performed by two reviewers, and four reviewers extracted data independently. Two additional reviewers performed the data revision and second check. The investigators collected the first author, procedure, location, study center, year of publication, study design, number of patients, and baseline characteristics (age, weight, cancer type) across study arms (Apixaban and LMWH). Primary endpoint outcomes included major bleeding, defined as bleeding requiring transfusion and fatal bleeding, CRNMB, and recurrent VTE. Secondary endpoint outcomes included DVT, PE, and all-cause mortality. Two reviewers assessed each of the included studies for bias using the Cochrane Risk of Bias 2.0 quality assessment tool [11].

Statistical Analysis

This meta-analysis followed the guidelines of the Cochrane Collaboration and Meta-analysis for observational studies in epidemiology. The Review Manager program, version 5.4.1 (Cochrane Foundation), was used for statistical analyses. For the binary outcomes in this study, we used the Mantel-Haenszel fixed-effects models to calculate the risk ratio (RR) and 95% confidence intervals (CIs) and the inverse variance approach to get the weighted mean difference for the continuous outcomes. To examine statistical heterogeneity, we used the Q-test for heterogeneity (Cochran 1954) and the I² statistics, with I² >50% denoting significant heterogeneity. A statistically significant outcome was defined as a p-value less than 0.05. A sensitivity analysis was used to verify the validity of the data, and the results were presented.

Results section

Included Studies and Baseline Characteristics

The sum of patients included across all four studies was 1632 adults with concomitant cancer and VTE. In all four of the included studies [12-15], the patients in the intervention group received 10 mg of Apixaban twice daily for the first seven days, followed by 5 mg daily by mouth. The patients in the control groups were placed on different regimens either Enoxaparin 1 mg/kg/SC every 12 hours [13] or dalteparin 200 IU/kg once daily for the first month followed by 150 IU/kg once daily [12,14,15].

Two of the analyzed studies included patients with different types of cancer, including colorectal, lung, breast, genitourinary, pancreatic/hepatobiliary, gynecologic, upper gastrointestinal, and hematological [14,15]. Another study excluded lung, hematological, or upper gastrointestinal cancers from the aforementioned list [13]. Notably, the fourth study uniquely limited its inclusion criteria to patients with only gastrointestinal, pancreatic, or hepatobiliary cancers [15]. The study characteristics are depicted in Tables 1, 2*.*

**Table 1:** Summary of studies included in the meta-analysis LMWH: Low Molecular Weight Heparin, CA-VTE: Cancer-Associated Venous Thromboembolism, ​​​​​​​DOACs: Direct Oral Anticoagulants, ​​​​​​​GI-Tract: Gastrointestinal tract

S. No	Study ID	Location	Study Center	Study Duration (year-year)	Study Design	Number of Patients		Study Conclusion
						Apixaban	LMWH	
1	Agnelli et al., 2020 [14]	Europe	Clinical Research Unit of the University of Perugia	2017-2019	Randomized Controlled Trial	576	579	Oral apixaban was noninferior to subcutaneous dalte­parin for the treatment of recurrent venous thromboembolism in patients with cancer. The frequencies of major bleeding were similar with apixaban and dalteparin, including major gastrointestinal bleeding.
2	Mokadem, 2020 [13]	Egypt	Beni-Suef University hospital	2020	Prospective randomized clinical study	50	50	No difference in major bleeding, minor bleeding or recurrent deep venous thrombosis in patients with active malignancy when treated with either apixaban or LMWH
3	McBane II et al., 2019 [15]	USA	Mayo Clinic Cancer Center	2015-2017	Randomized Controlled Trial	150	150	Oral Apixaban was associated with low major bleeding and VTE recurrence rates for the treatment of VTE in cancer patients.
4	Kim et al., 2022 [12]	Korea	Asan Medical Center	2021-2022	Randomized controlled trial	44	46	DOAC therapy further increased the risk of bleeding compared with dalteparin in patients with active advanced upper GI tract, hepatobiliary, or pancreatic cancer, suggesting that extra caution should be taken when selecting anticoagulants for CA-VTE.

 

**Table 2:** Baseline cancer types in patients receiving either Apixaban or LMWH in included studies

Cancer type	Study ID								
	Agnelli et al., 2020 [14]		Kim et al., 2022 [12]		McBane II et al., 2019 [15]		Mokadem et al., 2020 [13]		
	Apixaban (N = 531)	Dalteparin (N =542)	Apixaban (N = 44)	Dalteparin (N= 46)	Apixaban (N = 136)	Dalteparin (N= 132)	Apixaban (N = 50)	Dalteparin (N = 50)	
Colorectal	121	113	-	-	18	29	23	19	323
Lung	105	95	-	-	32	19	-	-	251
Breast	79	76	-	-	16	12	7	4	194
Genitourinary	66	73	-	-	13	14	10	9	185
Pancreatic/hepatobilliary	44	43	17	22	23	24	2	4	179
Gynecologic	60	59			14	15	8	14	170
Upper gastrointestinal	23	31	27	24	7	4	-	-	116
Hematological malignancy	33	52	-	-	13	15	-	-	113
	531	542	44	46	136	132	50	50	1,531

Risk of Bias Assessment

**Figure 2:**
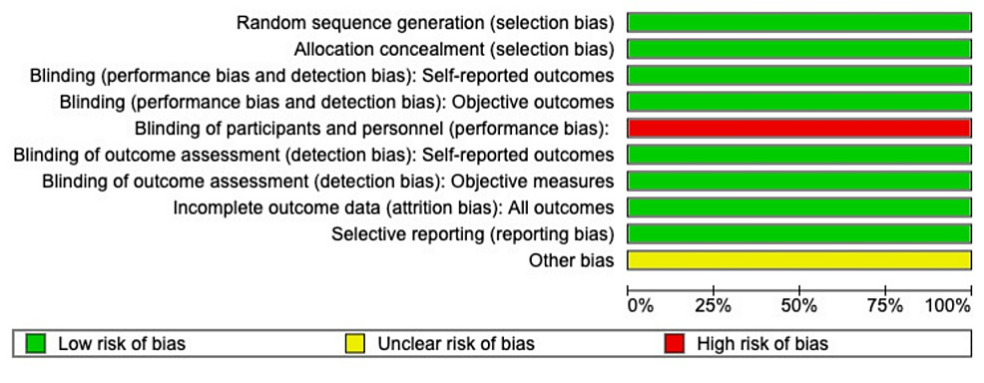
Risk of bias graph

**Figure 3:**
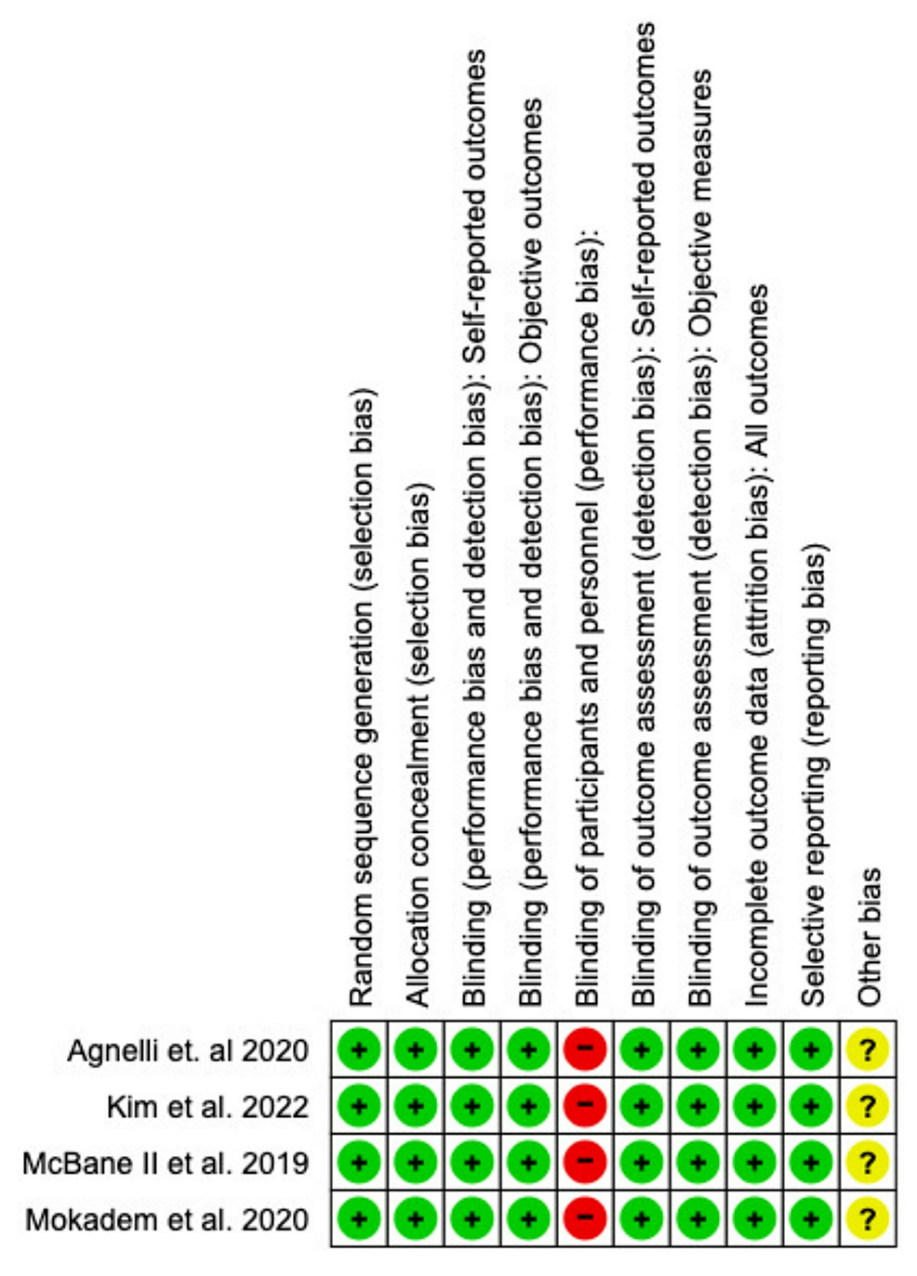
Risk of bias summary

The Cochrane Risk of Bias 2.0 RevMan quality assessment tool was used to construct the risk of bias graph and risk of bias summary depicted in Figures 2, 3, respectively. The studies included in this meta-analysis were found to have a low risk of bias in almost all domains except for a high risk of bias in the blinding of participants and personnel. All four studies were non-blinded due to the differences in the administration route of Apixaban (orally) versus LMWH (subcutaneously) [11].

*Primary Endpoint Outcome*s

**Figure 4:**
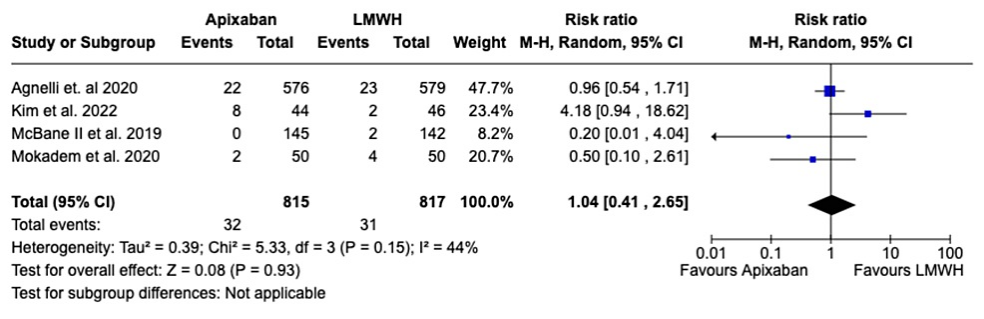
Relative risk for major bleeding with Apixaban v LMWH LMWH - Low Molecular Weight Heparin

**Figure 5:**
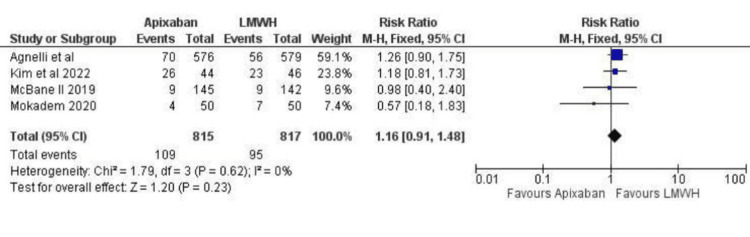
Relative risk for “Total Bleeding” (major bleeding and CRNMB) with Apixaban v LMWH CRNMB - Clinically Relevant Non-Major Bleeding, LMWH - Low Molecular Weight Heparin

**Figure 6:**
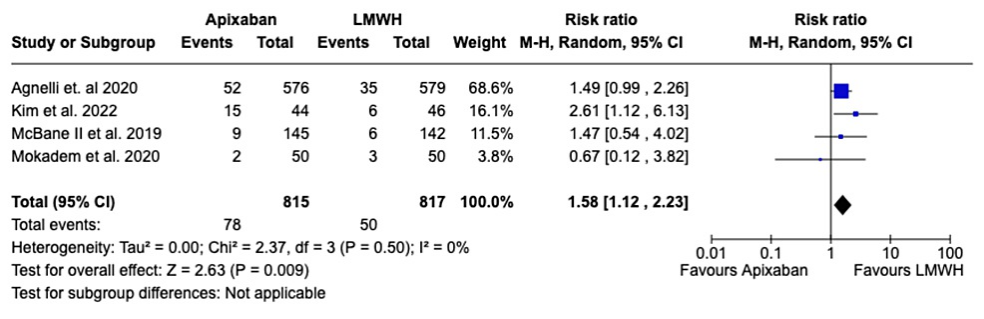
Relative risk for CRNMB with Apixaban v LMWH CRNMB - Clinically Relevant Non-Major Bleeding, LMWH - Low Molecular Weight Heparin

Based on the data from the four studies, the increase in the risk of major bleeding was not significantly different between the apixaban group versus the LMWH group (RR 1.04; 95% CI (0.65, 1.68); p=0.87; I^2^=44%) as is depicted in Figure 4. However, Apixaban was associated with a statistically significant increase in minor bleeding compared to LMWH (RR 1.57; 95% CI (1.12, 2.21); p=0.009; I^2^=0%), as depicted in Figure 5. There was, however, no significant difference between Apixaban and LMWH in terms of total bleeding (RR: 1.16; 95% CI (0.91,1.48); p=0.23; I^2^=0%), as depicted in Figure 6.

Secondary Endpoint Outcomes

Regarding recurrent venous thromboembolism, there was a statistically significant difference between the Apixaban and LMWH groups (RR: 0.61; 95% CI (0.41, 0.92); p=0.02; I^2^=7%), as depicted inFigure 7. Based on the three studies that reported recurrent DVT “any” there was no statistical difference between apixaban and LMWH (RR: 0.62; 95% CI (0.34, 1.14); p=0.24; I^2^=31%), as depicted in Figure 8. Apixaban was associated with a lower risk of recurrent PE when compared to LMWH. However, the results were of borderline significance (RR 0.58; 95% CI (0.34, 1.01); p=0.05; I^2^=0%), as depicted in Figure 9. Based on data from the 1,632 adults with cancer, there was no statistically significant difference between apixaban and LMWH in terms of mortality (RR: 0.89; 95% CI (0.73, 1.09); I^2^=0), as depicted in Figure 10. After assessing each I² statistic, it was concluded that there was no significant heterogeneity between studies.

**Figure 7:**
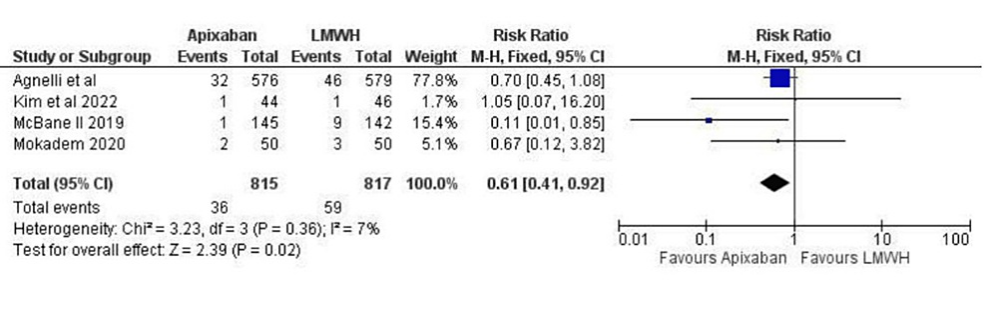
Relative risk for Recurrent VTE “Any” (DVT + PE) with Apixaban v LMWH VTE - Venous Thromboembolism, DVT - Deep Venous Thrombosis, PE - Pulmonary Embolism, LMWH - Low Molecular Weight Heparin

**Figure 8:**
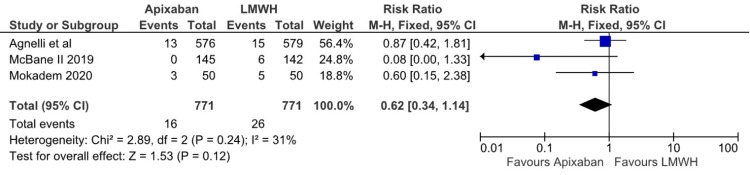
Relative risk for Recurrent DVT “Any” with Apixaban v LMWH DVT - Deep Venous Thrombosis, LMWH - Low Molecular Weight Heparin

**Figure 9:**
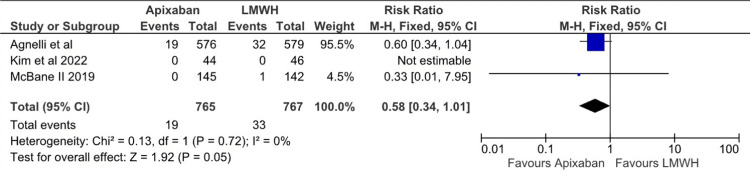
Relative risk for Recurrent PE with Apixaban v LMWH PE - Pulmonary Embolism, LMWH - Low Molecular Weight Heparin

**Figure 10:**
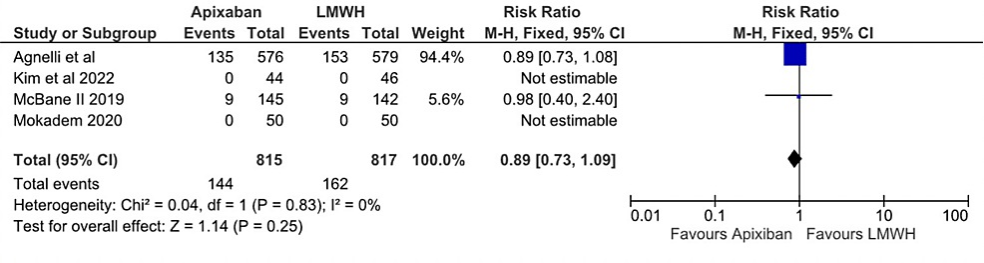
Relative risk for all-cause mortality with Apixaban v LMWH LMWH - Low Molecular Weight Heparin

Discussion

According to the CHEST Guideline and Expert Panel Report: Direct Oral Anti-Coagulants have been established as the recommended treatment for VTE in the general population [16]. Patients with cancer have a substantially higher risk of recurrent VTE despite anticoagulation therapy [17]. Recently, many RCTs have been conducted to compare the efficacy of DOACs with LMWH in treating CA-VTE and illustrated conflicting results regarding bleeding risks [18]. Hence, the question of the overall benefit-RR of DOACs vs. LMWH for VTE therapy in cancer patients persists.

This systematic review and meta-analysis analyzed data from four RCTs that compared the efficacy and safety of Apixaban with LMWHs for CA-VTE. The primary end-point outcomes included major bleeding, minor bleeding, and total bleeding. Based on the cumulative results of the four studies analyzed, there was no statistically significant difference between the risk of Major Bleeding and Total Bleeding in the group of patients taking Apixaban and those taking LMWH. The risk of Minor bleeding was higher in patients taking Apixaban than those taking LMWH. These results are comparable with Li et al.'s study that reported a higher risk of bleeding in cancer patients managed with DOACs than LMWH [19].

The secondary outcomes include recurrent VTE, recurrent DVT, recurrent PE, and all causes of mortality. The RR for recurrent VTE was 0.61, which indicates a lower risk in patients taking Apixaban. While considering recurrent DVT, the two groups had no statistically significant difference. Regarding recurrent PE, Apixaban was associated with a lower risk than LMWH. In terms of all-cause mortality, there was no statistically significant difference between the two groups.

According to the study conducted by Kim et al., major bleeding occurred in 18.2% of the patients in the DOAC group and 13% in the dalteparin group, and the GI tract was the most common site of bleeding. 2.3% of the patients in the DOAC group and 2.2% in the dalteparin group experienced recurrent CA-VTE. This study demonstrated that in patients with active advanced upper GI tract, hepatobiliary, or pancreatic cancer, DOAC treatment raised the risk of bleeding even more than dalteparin [12]. Most of the patients included in the clinical trial (42%) conducted by Mokadem et al. had colon cancer. The results displayed non-significant differences between both groups (Apixaban and LMWH) regarding Major and Minor bleeding, recurrent DVT, and VTE [13]. The results of this study are homogenous with Agnelli et al. In the Caravaggio trial [14], one-third of the patients presented with gastrointestinal cancer and an increased risk of major GI bleeding; 1.9% in the Apixaban group and 1.7% in the dalteparin group. The results of this study demonstrated that Apixaban was non-inferior to subcutaneous dalteparin in terms of recurrent VTE in patients with cancer. However, the risk of major bleeding was similar between the two groups, in contrast to the previous studies that showed a more significant risk with DOACs [13,14].

In the ADAM-VTE trial [15], oral Apixaban gained precedence over dalteparin in terms of both major bleeding and recurrent VTE. Unlike dalteparin, two out of 142 (1.4%) patients experienced major bleeding; no bleeding events occurred in the Apixaban group. Recurrent VTE occurred in 0.7% of the patients in the Apixaban arm and 6.3% in the dalteparin arm. This trial favors Apixaban as a suitable treatment regimen for CA-VTE [15].

Regarding the limitations of the included studies: patients with brain tumors were excluded from the Caravaggio study [14] due to safety reasons. Additionally, GI bleeding reported was not a prespecified outcome, and their trial was not powered to make a definitive conclusion about the risk of bleeding since it primarily focused on recurrent VTE [14]. The ADAM-VTE trial [15] had a relatively small study population of 300 patients. Moreover, it failed to achieve its predefined primary outcome to estimate the risk of major bleeding, due to very few major bleeding events [15]. Furthermore, secondary endpoints were not accounted for in the Mokadem study due to a small population size [13]. Similarly, the patients included in the study by Kim et al. failed to draw a meaningful comparison between the two interventions due to the small population size and numerically different cancer types. These contradictory bleeding risk outcomes might be explained in part by the studies' heterogeneous cancer types and disease states.

Moreover, GI cancer, particularly upper GI tract or pancreaticobiliary malignancies, is widely known for having the greatest VTE and bleeding risk, independent of anticoagulant administration [20,21]. A recent study at the Mayo Clinic using prospectively collected data from 1,392 CA-VTE patients found that apixaban had a higher rate of MB in GI tract cancer patients compared to non-GI tract cancer patients [22]. It is also observed that the bleeding episodes may be caused by GI mucosal damage caused by chemotherapy for GI tract cancer, along with high DOAC concentrations in the GI lumen [23,24]. Additionally, Caucasians have a slightly higher incidence of CA-VTE than Asians [25,26].

The comprehensive analysis of the four studies included in this meta-analysis provides substantial evidence supporting the non-inferiority of DOACs, specifically Apixaban, compared to LMWH in the context of cancer patients. While acknowledging certain limitations in relation to GI malignancies and an increased risk of minor bleeding, the overall findings indicate that DOACs can be considered a viable alternative to LMWH for anticoagulation in this patient population. However, it is essential to exercise caution and consider individual patient characteristics, such as age, cancer type, medical history, and bleeding risk, before making treatment decisions. Despite the promising results, further research and clinical trials are warranted to establish the optimal anticoagulation approach in cancer patients, ensuring the highest level of efficacy and safety for each individual.

## Conclusions

When selecting anticoagulant medication for CA-VTE, clinicians must thoroughly assess various patient factors, including cancer type, disease stage, overall health status, renal function, potential drug interactions, and patient preferences. The available evidence from this meta-analysis supports the non-inferiority of DOACs, particularly Apixaban, when compared to LMWHs in this context. However, it is crucial to acknowledge the existing limitations, such as variations in GI malignancies and a potential increased risk of minor bleeding. To establish more robust evidence regarding the effectiveness and safety of DOACs over LMWHs in CA-VTE, further extensive research is required. Ongoing research efforts and activities are necessary to address these gaps and provide more concrete guidance for clinical practice, enhancing treatment techniques and optimizing care for cancer patients with VTE.
